# Notes of Observations on the Character of the Red Blood Corpuscle

**Published:** 1867-06

**Authors:** Rufus King Browne


### ARTICLE II.

*Notes of Observations on the Character of the Red Blood Corpuscle.* By Professor Rufus King Browne, M.D.

There can be no doubt that it is a valid instinct in man, which has always assigned a commanding, if not supreme importance, in the complex processes of physiology, to the blood, and especially its red corpuscles. I have myself long been persuaded that all of the variations in these complex processes, which on the one side constitute health, and on the other disease—which two are only *divided* aspects of one and the same system, series, or sum of the animal functions,—will eventually be found to turn upon some change at a given time and under peculiar conditions, in either the rate of its progress, or the character of its main elements.

Accordingly, I have during several years sought on many occasions, to ascertain the true anatomical characters and then deduce the career, of the red blood corpuscle.

It is to this latter point that I propose briefly to ask attention, conscious that my observations, even with the highest known powers of the microscope, though repeatedly made very many times, have not as yet enabled me to affirm anything with entire certainty, yet I shall venture briefly to present the results of those observations.

The red blood corpuscle is regarded as an enduring anatomical element, but I am confident that they are the least so of any anatomical form in the body. I am confident, though as a *demonstration* my observations would fail to establish that conviction in the minds of others, that they are perpetually solved in the liquid sanguinis, and as constantly renewed.

This view is of course equally opposed to the notion on the one hand that they are permanent anatomical forms, and on the other, that they are "destroyed" in any special organ of the body.

The first striking feature of continued observation of these bodies, with *high* powers of the microscope, and without crushing them under the pressure of a covering glass, is that the *size* of each one of any given number of them varies between wide extremes, some being from three to four times the size of others. This great disparity is of course not sufficiently palpable to arrest the attention of the observer except under very high powers.

The opinion generally received is that the human red blood corpuscle is either a cell with red contents, the nucleus of which has disappeared, or that it is a free nucleus of a cell, and which, is not settled.

Neither of these views appear to me to accord with the result of careful observations. It consists of matter of different degrees of density in different parts, being much more nearly fluid in the interior than the outside. To this fact is attributable the depressed centre, which is not however equally visible in all red corpuscles. It has been most generally described as having a cell-wall, after the fixed stereodox form of designation of all such minute anatomical forms ; and this, long after the old and erroneous notion of a cell as a body having a triplex structure, in three distinct parts, as cell "wall," "nucleus" and "nucleolus" has been abandoned by the higher order of observers with the microscope.

When placed in some liquids, many of the corpuscles swell up and disappear, but in such cases I have never been able to detect any remaining cell substance.

Dr. Beale has shown conclusively that the existence of this cell-wall must be abandoned as rather a theoretical notion than an ascertained or observed fact, although many observers have asserted the bursting of the corpuscle and the escape of its contents. Guinea pig's blood crystalizes very rapidly in tetahedral crystals, and if the process be carefully watched in a drop of blood which has been treated with a very little water, (and sometimes without the water,) certain corpuscles will be seen to become angular, and four or eight prominent angles will be apparent, while others will exhibit the well-known stellate appearance. Afterward, the entire blood corpuscle will be seen to crystalize, and subsequently the crystals will be seen to coalesce and form larger crystals. Certainly no membrane nor anything resembling it exists here.

The red blood corpuscle from the same animal differs in character considerably. Some are darker and harder than others ; some are so transparent as to be nearly, if not totally, invisible, and some are not more than one-fourth the size of others. These facts, which appear to have escaped the attention of observers, are only applicable to the supposition that different corpuscles are in different stages of *growth* and progress.

Experiments with coloring matter strongly confirm this conclusion. The white corpuscles of the blood can be very readily tinged with a solution of carmine and glycerine. The granular or nucleated corpuscle of the embryo is also easily colored, while the majority of the red corpuscles cannot be colored by the same means. Nevertheless, in certain instances, some of the smaller corpuscles in the capillaries can be readily colored. These, however, are very much smaller than the white corpuscles, and do not present their granular appearance. *They are undoubtedly young red corpuscles.* There are, however, other observed facts which can lead to no other conclusion.

In winter, the capillaries of the common frog contain numerous oval corpuscles, not more than half the dimensions of the corpuscle when the same animal is active. In this case at least there can be no doubt of the fact that the former are *young* corpuscles. In the lymph canal, there may always be seen in the intervals of digestion small corpuscles of a similar character, and these may also readily be stained.

These observations lead me to the conclusion that the red blood corpuscle is either the separate form of the aggregations of rounded particles, apparently granular, forming the white corpuscle, or of the corpuscles I have designated found in the lymph canals, and that it undergoes a series of changes, by which it finally becomes converted into matter soluble in the plasma, and that they are thus continually dissolved and replaced.

